# Effects of different pre-conditioning exercise on leptin synthesis and its downstream signalling pathway in T2DM rats

**DOI:** 10.22038/ijbms.2024.77774.16828

**Published:** 2025

**Authors:** Sen Lin, Yuzhi Hu, Shuqiao Ding, Yazhe Hu

**Affiliations:** 1 School of Sports Science and Technology, Department of Sports Health, Wuhan Sports University, Wuhan, 430079, China; 2 School of Acupuncture-Moxibustion and Tuina, Beijing University of Chinese Medicine, 100029, Beijing, China; 3 School of Physical Education, Department of Sports Health, Central China Normal University, Wuhan, 430079, China

**Keywords:** Diabetes Mellitus, High-intensity interval training, Leptin, Lipid metabolism, Sports medicine

## Abstract

**Objective(s):**

This study aimed to evaluate the effects of pre-conditioning exercise on body lipid metabolism, leptin secretion, and the downstream pathways at the early stage of type 2 diabetes mellitus (T2DM).

**Materials and Methods:**

The T2DM model was established using an 8-week high-sugar, high-fat diet combined. The T2DM model was established using an 8-week high-sugar, high-fat diet combined with streptozocin (STZ) injection. Two exercise interventions, high-intensity interval training (HIIT) and moderate-intensity continuous training (MICT) were performed during the model-building process. One week following the STZ injection, rats were euthanized. Blood, gastrocnemius muscle, and epididymal fat pad were collected. Plasma leptin content was measured by ELISA. The expression of leptin-mRNA in epididymal adipose tissue was measured using RT-qPCR, and its protein expression was detected by a western blot. Leptin, leptin-R, and AMPK (AMP-activated protein kinase) - ACC (Acetyl-CoA carboxylase) expression in gastrocnemius muscle was also detected by western blot. Free fatty acids (FFA) and triglycerides (TG) contents in gastrocnemius muscle were measured using a biochemical assay.

**Results:**

In the HIIT group, glucose tolerance and leptin receptor expression increased, as did the expression and phosphorylation of AMPK protein. At the early stage of T2DM, it increased significantly in the gastrocnemius muscle in the MICT group.

**Conclusion:**

At the early stage of T2DM, pre-conditioning exercise in the form of HIIT was found to inhibit the leptin-mRNA expression in adipose tissue, suppress leptin synthesis, up-regulate AMPK-ACC signaling pathway, and promote lipid decomposition in skeletal muscle tissue. Pre-conditioning of MICT led to the accumulation of FFA and TG in skeletal muscle, likely due to exercise adaptation rather than ectopic deposition of lipids.

## Introduction

DM (diabetes mellitus) has become increasingly prevalent worldwide, with the number of affected individuals expected to reach 642 million by 2040 (1). T2DM, a common and obesity-related disease, is characterized by insulin resistance and pancreatic β cell dysfunction, and it has been challenging to control T2DM with current drug treatments (2, 3). In recent years, leptin has gained attention as a research focus in DM treatment due to its significant impact on reducing body weight and blood sugar levels.

Current evidence suggests that leptin plays a role in regulating energy balance by influencing lipid and glucose metabolism. Leptin promotes the oxidation of fatty acids in the liver, skeletal muscle, and pancreas, redirecting them away from triglyceride storage. It also prevents fat deposition in non-adipose tissues and increases insulin sensitivity in muscle and adipose tissue (3). Leptin selectively increases the activity of AMPK in skeletal muscle, and the AMPK-ACC signaling pathway is crucial for energy metabolism. Dysfunction in the Leptin-AMPK-ACC signaling pathway may contribute to metabolic abnormalities in T2DM (4, 5). In diabetes, AMPK activity is inhibited, leading to disrupted energy metabolism (6). However, exercise has been shown to improve skeletal muscle’s glucose and lipid metabolism through AMPK activation (7). 

Exercise has gained increasing attention as an important strategy for treating DM. Numerous studies have demonstrated that exercise improves lipid metabolism in diabetic rats by improving leptin resistance (8, 9). Specifically, MICT has been shown to improve impaired glucose tolerance and insulin resistance in diabetic patients, while HIIT, characterized by intermittent periods of effort followed by recovery periods, has been found to yield more significant improvements in insulin sensitivity and higher adherence compared to MICT (10, 11). However, limited studies have focused on the effects of different pre-conditioning exercises on leptin and its downstream pathways in diabetic patients. In this study, exercise interventions were utilized during the establishment of a T2DM model to investigate the potential impact of pre-conditioning exercise on the secretion of leptin and its downstream pathways in rats after the onset of DM. Furthermore, the study aimed to examine the influence of exercise on body lipid metabolism and elucidate the mechanisms by which exercise can prevent the development of DM. 

## Materials and Methods

*Experiment scheme*

**Figure 1:**
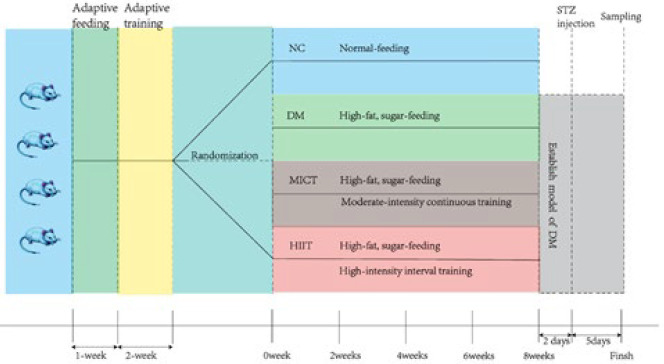
Experimental Process for SD Rats (Sprague-Dawley) is Shown in Figure 1

Fifty 8-week-old SD rats were randomly divided into four groups: NC (normal control), DM (diabetes mellitus), HIIT (high-intensity interval training), and MICT (moderate-intensity continuous training) groups, with ten rats in each group. After one week of adaptive feeding and two weeks of adaptive exercise training, rats were housed in a Specific Pathogen Free (SPF) animal laboratory at Wuhan Sports University (certificate number SCXK2015-0018, No.42000600025097). The NC group was fed a regular diet, while the DM, HIIT, and MICT groups were fed on a high-sugar, high-fat diet, which consisted of basic feed (64.5%), cane sugar (18%), lard (10%), egg yolk (5%), cholesterol (2%), and sodium cholate (0.5%). The experimental process is illustrated in Figure 1.

The HIIT and MICT groups were subjected to high-intensity interval running exercise and moderate-intensity running exercise, respectively, for 40 min every day and five days per week (12). For detailed exercise paradigms, please refer to the studies by Table 1 and Table 2.

On the 3rd day after the pre-conditioning exercise intervention, DM, HIIT, and MICT groups were intraperitoneally injected with a 2% STZ (Sigma, USA) solution at a dose of 30 mg/kg, while the NC group received the same amount of citric acid buffer. IPGTT was performed five days later. At the end of the 9^th^ week, after euthanasia by carbon dioxide asphyxiation, blood is taken from the heart within two minutes. Rats with random blood glucose (RBG) above 250 mg/dl are considered diabetic (13). Gastrocnemius muscle and epididymal fat were collected, washed with normal saline, weighed, and frozen. Plasma leptin content was measured using ELISA, epididymal fat leptin-mRNA was measured by RT-qPCR, leptin protein content in adipose tissue of rat epididymis and leptin and leptin-R, AMPKα/P-AMPKα, ACC/P-ACC protein expression in gastrocnemius muscle were detected by western blot. Additionally, FFA and TG contents in gastrocnemius muscle were measured using an automatic biochemical analyzer.

*Body weight and intraperitoneal glucose tolerance test*

A glucose tolerance test was performed on the 7th day after the STZ injection. The rats were subjected to a 12-hour fasting period, followed by an intraperitoneal injection of glucose solution (2 g/kg). Blood samples were collected from the tail vein at 0 min, 30 min, 60 min, and 120 min post-injection, and blood glucose levels were measured. A blood glucose curve was plotted, and the area under the curve (AUC) was calculated.

***Quantitative real-time polymerase chain reaction***

The epididymal fat tissue was frozen, crushed in liquid nitrogen, and transferred into TRI pure tubes (Aidlab Biotechnology, Beijing, China) for RNA isolation and extraction. RNA integrity was determined by agarose gel electrophoresis. Complementary DNA (cDNA) was synthesized from RNA using the reverted RT kit (Thermo Fisher, Waltham, MA, USA) according to the manufacturer’s instructions. cDNA was mixed with forward and reverse primers of leptin in a 96-well plate containing AceQ Universal SYBR qPCR Master Mix (Vazyme Biotech, Nanjing, JS, China). The primer sequences used in the experiments are designed by Sangon Biotechare (Shanghai, China) and are shown in Table 3. The analysis was done using the StepOnePlus Real-Time PCR System(Applied Biosystems, Waltham, MA, USA). The detailed conditions for RT-qPCR (temperature and time in each step, number of cycles, and melting curve ) were shown in supplementary material 1. The relative gene expression (RQ) in each sample was calculated with reference to the control sample using the following formula: RQ= 2-ΔΔCt.

*Western blot*

Leptin, leptin-R, AMPKɑ, P-AMPKɑ, ACC, P-ACC in gastrocnemius muscles, and leptin in the epididymal fats expression were measured by western blot. Tissues were lysed with RIPA buffer (Beyotime #P0013B) containing 1mM PMSF (Amresco #0754). The homogenates were then spun at 12000 (relative centrifugal force (RCF) ×g) for 10 min at 4 ^°^C. The supernatants were collected, and the protein concentrations were determined using the BCA Protein Assay reagent (Aidlab BiotechnologiesCo., Ltd #PP0102).

The samples were diluted with 5× loading buffer and separated on a 10% SDS-polyacrylamide gel by electrophoresis at 100 V. Then the proteins were transferred onto polyvinylidene fluoride (PVDF) membranes. The membranes were blocked for 60 min in TBST containing 5% nonfat milk and then incubated overnight with the primary antibodies (Table 4).

The membranes were then incubated with the appropriate anti-rabbit secondary antibody and diluted in TBST. The antibody dilution for the western blots was as per the manufacturer’s recommendation. According to the manufacturer’s instructions, the proteins were visualized using the ECL west Blot Detection Kit (Beyotime, P0018, China). Band quantification was determined using the Quantity One 4.62 Gel optical density analysis software (Bio-Rad, Hercules, CA, USA).

*TG and FFA measurement in muscle*

To prepare a 10% tissue homogenate from 100 mg muscle tissue, the tissue was mixed with 1 ml pre-cooled physiological saline and homogenized thoroughly using a homogenizer. The homogenate was spun at 3300 (relative centrifugal force (RCF) ×g) for 10 min, and the resulting supernatant was collected for further testing. The detection of TG and FFA is used for the Triglyceride (TG enzyme method) test kits (Jian Cheng Bioengineering Institute, F001-1-1, Nanjing, China) and the Non-esterified Free Fatty Acids assay kit (JianCheng Bioengineering Institute, A042-1-1, Nanjing, China).

***Statistical analysis***

SPSS 20 statistical software (SPSS Inc., Chicago, IL, USA) was used for data analysis. The data were presented as mean±standard deviation (SD). Significance among different groups was determined by using the one-way ANOVA test with Tukey’s *post hoc *test. *P*-values<0.05 indicated statistical significance.

## Results

*Body weight and gastrocnemius muscle and epididymal fat pad weights*

The weight of the body, bilateral epididymal fat pad, gastrocnemius muscle weight, as well as the random blood glucose and insulin, were shown in Table 5. In T2DM modeling groups (DM, HIIT, and MICT), the body weights decreased significantly compared to the NC group (*P*<0.01). The bilateral epididymal fat pad weights in the NC and DM groups were significantly higher than in the MICT and HIIT groups (*P*<0.01, *P*<0.01). 

Skeletal muscle weight loss can be observed in the DM and MICT groups (*P*<0.01, *P*<0.01), while HIIT showed a trend towards alleviating this symptom (*P*=0.057). 

*Glucose tolerance test by intraperitoneal injection*

IPGTT was conducted to evaluate insulin sensitivity. AUC in the NC group was significantly lower than in the DM, MICT, and HIIT groups, which suggests impaired glucose tolerance in the DM, MICT, and HIIT groups compared with the NC group (*P*<0.01).

**Figure 2:**
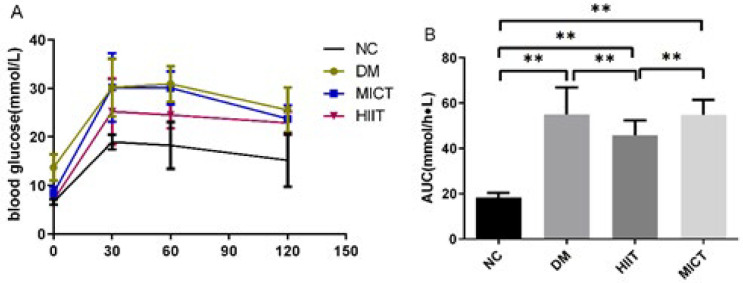
Plasma glucose during IPGTT and the area under the curve (AUC) for plasma glucose levels

In addition, high-intensity interval exercise pre-conditioning was found to significantly lower AUC in the HIIT group compared to those in DM and MICT groups, suggesting that it can improve impaired glucose tolerance in the early stage of DM (Figure 2).

*Changes in leptin synthesis and expression of leptin receptors in muscle*

**Figure 3:**
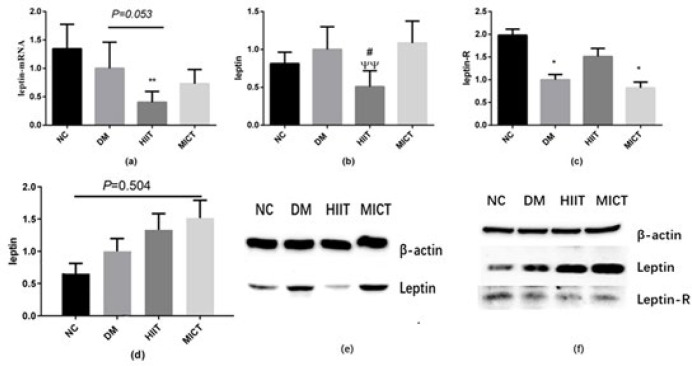
Evaluation of leptin and its receptors in adipose and gastrocnemius muscle tissues

Our results show that in the HIIT group, there was a tendency for lower mRNA expression of leptin (*P*=0.053) in adipose tissue, and there was a significant reduction in its protein expression compared to the DM group (*P<*0.05) (Figures 3a, b, and e). 

The expression of leptin receptors in muscle tissue significantly decreased in the DM group (*P*<0.05)(Figures 3c and f). The expression of leptin in muscle tissue exhibited an inclination towards an increase in the MICT group, compared to the NC group (*P*=0.0504) but no significant differences were observed in the expression of leptin protein in gastrocnemius muscle (Figures 3d and f). 

*AMPK-ACC protein expression levels in muscles*

**Figure 4:**
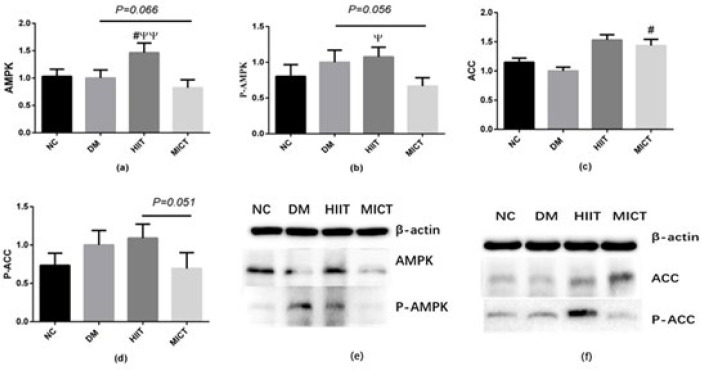
Relative expression of AMPKα and ACC proteins and their phosphorylation levels in muscle tissue

It has been shown that leptin can activate the AMPK-ACC signal pathway and promote the breakdown of fatty acids. Therefore, we examined the expression and phosphorylation of AMPK and ACC. Figure 4 demonstrates that compared to the DM group, there was a clear trend towards the inhibition of the expression and phosphorylation of AMPK𝛼 in the MICT group (*P*=0.066, *P*=0.056). In contrast, the expression of AMPK𝛼 in the HIIT group showed a significant increase (*P*<0.05). The expression of ACC in the MICT group was significantly increased (*P*<0.05).

*FFA and TG content in the muscles of rats in each group*

**Figure 5:**
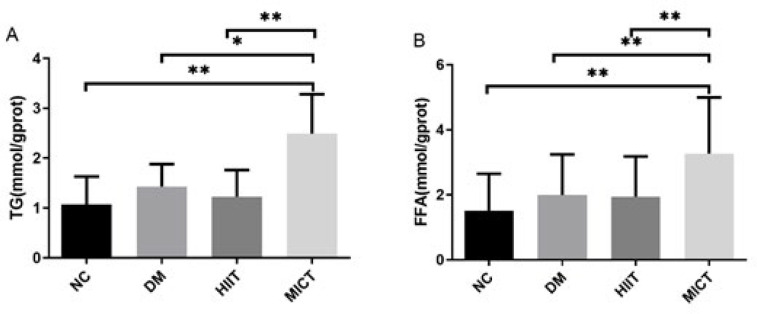
Triglycerides (TG) and free fatty acids (FFA) contents in muscle tissue of rats

To further investigate the lipid metabolism of skeletal muscle, the levels of TG and FFA in skeletal muscle tissue were measured. Figure 5 illustrates that the levels of FFA and TG in the MICT group were notably higher compared to those in the NC, DM, and HIIT groups. It suggests that MICT led to a significant increase in FFA and TG levels in muscles.

## Discussion

In this study, a high-sugar / high-fat diet was used to induce insulin resistance and leptin resistance in the animals. Additionally, a low-dose STZ injection was administrated to establish the T2DM animal model. This approach offers several advantages, including a short time frame, simple implementation, low cost, and relative reliability and stability (14)

Leptin is a single-chain protein hormone first found in adipose tissue, playing a crucial role in regulating energy metabolism (15). Insulin resistance (IR) and lipid metabolism disorders are often caused by leptin deficiency or dysfunction (1, 3). Adipose tissue is essential for energy storage in the body. White adipose tissue is no longer considered an inert tissue used primarily for energy storage; instead, it is now recognized as an active participant in regulating physiological functions (16). As an endocrine organ, adipose tissue is important for the development of many obesity-related diseases. It also plays a vital role in regulating circulating leptin levels as the primary source of leptin secretion (17, 18).

Insulin could increase leptin mRNA and leptin protein expression in adipose tissue (19). The DM group experienced the process of diabetes modeling; the islet β cells were destroyed by STZ, and the insulin level was reduced (20). Pieu found that leptin mRNA expressions were significantly increased in adipose tissue of non-obese type 2 diabetes mellitus (21). However, we found that, in adipose tissue, no significant difference in leptin mRNA/protein level was observed between the NC group and the DM group. This indicates that the leptin gene transcription or leptin protein synthesis in the early stage of T2DM rats was not affected. A limitation of this study is that more groups should be set up for a longer period of time after the development of diabetes.

Results indicated that pre-application of HIIT has a significant inhibitory effect on OB-gene transcription and protein synthesis in adipose tissue in the early stage of T2DM formation, but its specific mechanism requires further study.

Leptin is a well-known adipokine but has also been found to be expressed in skeletal muscle (22). Studies have shown that leptin mRNA can be detected in skeletal muscle at a level similar to that of subcutaneous fat (23). A study found that leptin can be secreted in human skeletal muscles, and the leptin release rates per 100g of adipose tissue and skeletal muscle are 0.8 ng/min and 0.5 ng/min, respectively (24). These findings suggest that skeletal muscle may play a significant role in leptin production in individuals with lower body fat, as muscle mass is relatively larger compared to fat. The expression of leptin protein in gastrocnemius muscle showed an upward trend in the MICT group. In the early stages of T2DM, there is no significant difference in the expression of leptin protein in the gastrocnemius muscle. 

However, there is limited research on the expression of leptin receptor protein in skeletal muscle tissue and its expression in different periods of DM. In this study, we found that the expression of receptor protein in muscle tissue increased in the MICT and DM group compared to the NC group. But there is no significant difference between MICT, HIIT, and DM groups. This indicates that the regulation of leptin receptor protein in muscle tissue may require longer exercise intervention. 

Different forms of exercise have distinct effects on leptin synthesis. HIIT reduces leptin expression in adipose tissue by inhibiting its transcription and its protein synthesis. However, HIIT and MICT do not reduce the expression of leptin protein in skeletal muscle. Skeletal muscle plays an important role in regulating metabolism during exercise (25). Leptin regulates skeletal muscle metabolism through the central system, promotes the oxidation of FA in skeletal muscle by activating AMPK and phosphorylating/inhibiting ACC, resulting in the decrease of malonyl CoA, and increases FA entry into mitochondria through carnitine palmitoyl transferase 1, thereby promoting FA oxidation. Kang *et al*. found that leptin increased the expression and activation (phosphorylation) of AMPK and phosphorylation of ACC through a two-week leptin intervention in rodents, indicating that leptin can activate the AMPK-ACC signaling pathway (26).

Increased circulating leptin may not accurately indicate skeletal muscle leptin resistance and its resistance may be related to the expression of skeletal muscle leptin receptors (27). Studies have reported that exercise training and dietary control can affect leptin sensitivity through the interaction of the hypothalamus, liver, and muscle tissue in high-fat diet-induced obese rats. Kang *et al*. found peripheral leptin resistance rather than central leptin resistance in high-fat diet-induced obese rats for 13 weeks (26). In line with this, our results showed that the expression level of leptin-R in DM and MICT groups was significantly reduced, suggesting that leptin resistance may appear in the body.

Andrew et al. discovered that both HIIT and MICT can activate ACC and that the phosphorylation level of ACC in exercise can reach 2.5 and 3 times that of the control group (28). In this study, we found no difference in AMPK and ACC protein expression and phosphorylation levels between NC and DM groups, suggesting that there may be no abnormalities in the Leptin-AMPK-ACC signaling pathway in the early stage of DM. HIIT activates the downstream AMPK-ACC signaling pathway to improve oxidative metabolism and exercise capacity in the skeletal muscle of obese rats (29). In our experiment, the protein expression level of AMPKα in the HIIT group increased significantly compared to the DM and MICT groups, while the phosphorylation level of AMPKα protein in the HIIT group was also significantly higher than that in the MICT group. Similarly, the phosphorylation level of ACC in the HIIT group was higher than that in the MICT group. All these indicate that HIIT may activate the AMPK-ACC signal pathway in skeletal muscle during the establishment of the T2DM rat model.

The reason for this may be that HIIT’s large energy demand results in a significant increase in cellular ADP/ATP and AMP/ATP ratios, which can activate AMPK. AMPK phosphorylates several key enzymes involved in regulating lipid and protein metabolism and glucose transport (30, 31). Previous studies have shown a positive correlation between AMPK phosphorylation in skeletal muscle and exercise intensity and duration (32). Although the total amount of exercise in the HIIT and MICT groups is the same, HIIT has a higher intensity within a short period of time, which might explain the increased expression and activation of AMPKα in the HIIT group. 

Previous studies have reported peripheral lipid accumulation in diabetic rats (32), while our results showed no difference in TG and FFA levels between the DM and NC groups. The difference in results may be due to the timing of the measurements. In the Sakul study, measurements were taken several weeks after the development of diabetes (32), while in this study, lipid levels were measured one week after STZ injection. Interestingly, there was a significant increase in TG and FFA levels in the MICT group. Combined with results from weighting epididymal fat, it suggests that the increase in skeletal muscle lipid content in the MICT group may be related to exercise intervention rather than lipid accumulation in the body. This has been considered an important energy reserve during exercise and is affected by exercise (33), thus referred to as “athlete’s paradox” (34). Taking into account the above analysis, it is confirmed that different exercise modes have different effects on the body’s lipid metabolism, and HIIT may better activate the AMPK-ACC signaling pathway. 

**Table 1:** Scheme for high-intensity interval running exercise: duration, frequency, and intensity parameters

High-intensity interval running exercise										
2min	5min	4min	5min	4min	5min	4min	5min	4min	2min	1060m
7m/min	18m/min	42m/min	18m/min	42m/min	18m/min	42m/min	18m/min	42m/min	7m/min	

**Table 2:** Scheme for moderate-intensity running exercise: duration, frequency, and intensity parameters

Moderate-intensity running exercise program			
3 min	34 min	3 min	1060m
18 m/min	28 m/min	18 m/min	

This study has been approved by the Animal Experiment Ethics Committee of Wuhan Sports University (DW-20170901) and follows the Guide for Care and Use of Laboratory Animals

NC: Normal control group;DM: Diabetes mellitus group; HIIT: High-intensity interval training group; MICT: Moderate-intensity continuous training group

**Table 3:** Primer sequences for leptin protein and actin in SD rats

**Target gene**		**Primer sequence**
Leptin	up	5’-TGTTCAAGCTGTGCCTATCCA-3’
	down	5’-GCCCGGGAATGAAGTCCAAA-3’
Actin	Up	5’-CGTTGACATCCGTAAAGACCTC-3’
	down	5’-TAGGAGCCAGGGCAGTAATCT-3’

**Table 4:** List of primary and secondary antibodies: sources and product models

**Primary antibodies**	**Company name**	**Product model**
Mouse anti-β-actin	Sungene Biotech	KM9001
Rabbit anti-AMPK	Cell Signaling Technology	5831
Rabbit anti-P-AMPK	Cell Signaling Technology	2535
Rabbit anti-Leptin	Boster Biological Technology	BA1231
Rabbit anti-Leptin-R	Proteintech group	20966
Rabbit anti-ACC	Proteintech group	21923
Rabbit anti-P-ACC	Abcam	ab68191
**Second antibody**	**Company name**	**Product model**
Goat Anti Rabbit IgG/HRP	Sungene Biotech	LK2001

**Table 5:** Effect of different exercise regimens on body weight, EFW, GW, RBG, and insulin levels

	NC	DM	MICT	HIIT
Body weight(g)	520.38±51.70	466.32±44.15^*^	427.74±35.01^**#^	438.97±35.57^**^
EFW(g)	8.85±1.28	7.75±1.96	3.94±1.31^**##^	3.96±1.02^**##^
GW(g)	3.02±0.25	2.51±0.12^**^	2.43±0.54^**^	3.01±0.48^Ψ^
RBG (mmol/L)	7.01±0.44	26.95±7.59^**^	31.20±2.93^**^	28.90±2.92^**^
Insulin (pmol/L)	74.71±36.19	96.07±35.49	78.18±23.69	85.10±29.67

Results were expressed as mean±standard error (n=10)

Differences between groups were compared by one-way analysis of variance (ANOVA). When compared with the NC group, *P<0.05, **P<0.01; when compared with the DM group, ^#^*P<*0.05, ^##^*P<*0.01; when compared with the MICT group, ^Ψ^*P<*0.05, ^ΨΨ^*P<*0.01

EFW: bilateral epididymal fat pad weight; GW: gastrocnemius muscle weight; RBG: random blood glucose; MICT: moderate-intensity continuous training; NC: normal control; DM: diabetes mellitus; HIIT: High-intensity interval training

A) Plasma glucose during IPGTT, B) Area under the curve of plasma glucose

*P<0.05, **P<0.01

a) Leptin-mRNA evaluation in adipose tissue. b) Leptin protein evaluation in adipose tissue. c) Leptin receptors’ relative expression evaluates in gastrocnemius muscle tissue. d) Detection of leptin in gastrocnemius muscle tissue. e) Leptin expression in adipose tissue. f) Gastrocnemius muscle leptin and leptin receptor expression

Results were expressed as mean ± standard error (n =10). *P<0.05, **P<0.01

When compared with the NC group, *P<0.05, **P<0.01; when compared with the DM group, ^#^*P<*0.05, ^##^*P<*0.05; when compared with the MICT group, ^Ψ^*P<*0.05, ^ΨΨ^*P<*0.01

a) Changes in AMPKα protein content; b) AMPKα protein phosphorylation level; c) Changes in AMPKα protein content in muscle tissue; d) ACC protein phosphorylation level; e) AMPKα and P-AMPKα; f) ACC and P-ACC.β-actin as an internal standard

Results were expressed as mean±standard error (n =10). *P<0.05, **P<0.01

When compared with the NC group, *P<0.05, **P<0.01; when compared with the DM group, ^#^*P<*0.05, ^##^*P<*0.05; when compared with the MICT group, ^Ψ^*P<*0.05, ^ΨΨ^*P<*0.01

*P<0.05, **P<0.01

## Conclusion

Pre-conditioning of HIIT can inhibit the leptin gene transcription in adipose tissue, thereby reducing leptin synthesis in adipose tissue and regulating the body’s leptin content. In the early stage of T2DM, a certain level of leptin resistance and insulin resistance can be observed, while the skeletal muscle Leptin-AMPK-ACC signaling pathway remains unaffected. During the modeling process of T2DM rats, the pre-condition of HIIT activates the leptin-AMPK-ACC signaling pathway in the skeletal muscle, thereby improving leptin resistance and the lipid metabolism abnormality. It is noteworthy that there is no significant lipid deposition in skeletal muscle. Pre-conditioning exercise of MICT can cause FFA and TG accumulation in skeletal muscle.

## Data Availability

The datasets used and/or analyzed during the current study are available from the corresponding author upon reasonable request.

## Declaration

We have not used any AI tools or technologies to prepare this manuscript.

## Limitations and Suggestion

More groups should be set up for a longer period of time after the development of diabetes. An exercise control group should be set.
